# Comparison of L L Polyethylene Glycol Plus Ascorbic Acid and Oral Sodium Sulfate Tablets for Colonoscopy Bowel Preparation

**DOI:** 10.3390/jcm13237493

**Published:** 2024-12-09

**Authors:** Jin Hwa Park, Minjun Kim, Seung Wook Hong, Sung Wook Hwang, Sang Hyoung Park, Dong-Hoon Yang, Byong Duk Ye, Seung-Jae Myung, Suk-Kyun Yang, Jeong-Sik Byeon

**Affiliations:** 1 Department of Gastroenterology, University of Hanyang College of Medicine, 222-1, Wangsimni-ro, Seongdong-gu, Seoul 04763, Republic of Korea; pjh6718@hanmail.net; 2 Department of Gastroenterology, University of Ulsan College of Medicine, Asan Medical Center, 88, Olympic-ro 43-gil, Songpa-gu, Seoul 05505, Republic of Koreasjmyung@amc.seoul.kr (S.-J.M.);

**Keywords:** bowel preparation, polyethylene glycol, ascorbic acid, oral sodium sulfate, tablet

## Abstract

**Background and Aims:** A low-volume (1 L) polyethylene glycol plus ascorbic acid (PEG-A) solution and an oral sodium sulfate tablet (OST) formulation are recently introduced agents for colonoscopy bowel preparation. This study investigated the efficacy, safety, and tolerability of 1 L PEG-A vs. OST. **Methods:** This single-center, prospective, randomized, endoscopist-blinded study randomly assigned patients into 2 groups: 1 L PEG-A (group A); and OST (group B). Efficacy of bowel preparation was evaluated using the Boston Bowel Preparation Scale (BBPS). Tolerability and safety were investigated with a standardized questionnaire. **Results:** A total of 174 patients were included in the final analysis (group A, n = 92; group B, n = 82). Successful bowel preparation was achieved in 91.3% and 95.1% of patients in groups A and B, respectively (*p* = 0.324). Overall mean satisfaction with bowel preparation was greater among those in group B vs. those in group A (8.2 ± 1.7 vs. 6.8 ± 2.0, respectively; *p* < 0.001). Although abdominal distension was less common in group A than group B (3/92 [3.3%] vs. 9/82 [11.0%], respectively; *p* = 0.045), overall adverse events developed similarly in both groups (27/92 [29.3%] vs. 21/82 [25.6%], *p* = 0.583). In subgroup analysis of older patients (≥65 years of age), efficacy, overall satisfaction, and safety profiles were not different between groups A and B. **Conclusions:** Both 1 L PEG-A and OST demonstrated efficacy, tolerability, and safety for colonoscopy bowel preparation. OST was slightly better tolerated, whereas 1 L PEG-A resulted in less abdominal distension. Both agents were effective and safe in older patients.

## 1. Introduction

Adequate bowel preparation is an important quality indicator for colonoscopy [1]. Inadequate preparation reduces the detection rate of colorectal neoplasia, including advanced neoplasia >10 mm in size [2,3], thereby increasing the risk for interval cancer. The ideal bowel preparation should quickly remove all fecal material for reliable colonoscopy observation without causing gross or histological changes to the colonic mucosa. In addition, bowel preparation should not cause patient discomfort and should be safe [4,5].

Polyethylene glycol (PEG) is a traditional bowel preparation agent. Although the standard 4 L PEG dosing regimen is effective and safe [6], patient compliance is poor due to the salty taste, unpleasant sulfate odor, and high intake volume. Therefore, low-dose PEG agents have been developed for better tolerability, which include 1–2 L PEG with ascorbate/ascorbic acid (PEG-A) [7]. Previous studies have reported that a 1 L PEG-A solution demonstrated a non-inferior bowel cleansing quality and adenoma detection rate in comparison with a 2 L PEG-A and magnesium citrate plus sodium picosulfate solution [8,9].

Oral sodium sulfate solution is a bowel preparation agent containing sulfates of sodium, magnesium, and potassium as active ingredients. Oral sodium sulfate solution is not inferior to 4 L PEG with regard to bowel preparation safety and efficacy [10,11,12]. In addition, oral sodium sulfate solution has demonstrated similarly high efficacy compared with 2 L PEG-A [13,14]. However, some patients still complain of poor taste and odor of the oral sodium sulfate solution.

An oral sodium sulfate tablet (OST) is a tablet formulation that has been newly developed to further improve compliance by removing the unpleasant odor of the solution while maintaining the same ingredients as the oral sodium sulfate solution. In a randomized controlled study, the OST formulation demonstrated a non-inferior bowel cleansing efficacy and similar adenoma detection rate compared with the 2 L PEG-A solution [15]. OSTs demonstrated no serious adverse events in this study. Despite the potential benefit of OSTs, additional studies directly comparing the OST formulation with the 1 L PEG-A solution—the lowest-dose regimen—are lacking. As such, the present study aimed to compare the efficacy, safety, and tolerability of the 1 L PEG-A solution vs. the OST formulation.

## 2. Materials & Methods

### 2.1. Study Design

This prospective, randomized, investigator-blinded, controlled study compared 1 L PEG-A solution with the OST formulation. The PEG-A formulation (CleanViewAL powder, Taejoon Pharm Co., Ltd., Seoul, Republic of Korea) is composed of polyethylene glycol 3350, anhydrous sodium sulfate, potassium chloride, sodium chloride, and ascorbic acid. The OST formulation (Orafang, Pharmbio Korea Inc., Seoul, Republic of Korea), currently available only in the Republic of Korea, consists of 28 tablets containing sodium sulfate, potassium sulfate, magnesium sulfate, and simethicone.

Asymptomatic adults ≥18 years of age, who underwent colonoscopy at the Department of Gastroenterology at Asan Medical Center (Seoul, Republic of Korea), were enrolled. The main indications for colonoscopy were screening and surveillance for colorectal neoplasia. Symptomatic individuals who underwent diagnostic colonoscopy for evaluation of lower gastrointestinal symptoms and signs, such as chronic diarrhea and abdominal pain, were also included in this study. Polypectomy was performed if polyps were detected during colonoscopy. Individuals with colorectal diseases, such as inflammatory bowel disease and colorectal cancer, those with distinct hematochezia/melena, renal dysfunction (chronic kidney disease stage IV or V), a history of colorectal surgery, and those who refused enrollment, were excluded from the study. A total of 184 patients were initially enrolled in this study for 6 months, starting in February 2021. Patients were randomly assigned in a 1:1 ratio to a 1 L PEG-A or an OST group (groups A and B, respectively) using a computer-generated randomization code. Endoscopists were blinded to the randomization results. A total of eight board-certified gastrointestinal endoscopists, each with experience performing more than 500 colonoscopies, participated in this study. To prevent the endoscopists from being a confounding factor affecting tolerability and patient satisfaction, they participated evenly in performing colonoscopies in both groups.

This study has been registered in the Clinical Research Database (CRIS registration number: KCT0006143) and approved by the Clinical Review Board of Asan Medical Center (Institutional Review Board approval number 2020-1122).

### 2.2. Bowel Preparation Protocol

Group A self-administered 500 mL of PEG-A solution with 500 mL of water the evening before colonoscopy for those undergoing morning colonoscopy. On the morning of the colonoscopy, an additional 500 mL PEG-A solution with 500 mL water were administered. For those in group A undergoing afternoon colonoscopy, 500 mL of PEG-A solution with 500 mL of water were administered on the morning of colonoscopy; then, an additional 500 mL of PEG-A solution and 500 mL of water were administered 1–2 h later. This preparation was completed at least 2 h before the beginning of colonoscopy.

Group B self-administered 14 OSTs and 425 mL of water the evening before colonoscopy for those undergoing morning colonoscopy. Patients took 425 mL of water twice more for the following 1 h. On the morning of colonoscopy, an additional 14 OSTs were administered with 425 mL of water, followed by two more doses of 425 mL of water over the next hour. For those in group B undergoing afternoon colonoscopy, 14 OSTs with 425 mL of water were taken, followed by an additional 425 mL of water twice for 1 h. After 1–2 h, another 14 OSTs were taken with 425 mL of water, followed by two additional doses of 425 mL of water over the next hour. OSTs and additional fluid intake were completed at least 2 h before colonoscopy.

### 2.3. Study Endpoints

The primary interest was bowel preparation efficacy, evaluated using the Boston Bowel Preparation Scale (BBPS) [16]. Representative photographs depicting each BBPS score (0–3) were placed on the walls of the colonoscopy rooms for real-time reference. Bowel preparation success was defined as a BBPS score ≥ 2 for each colon segment and a total BBPS score ≥ 6.

Secondary endpoints were tolerability and safety parameters, which were evaluated using a standardized questionnaire. Overall satisfaction with the preparation was evaluated on a scale of 0 to 10 (higher score = higher satisfaction). Willingness to use the same agent for the next colonoscopy was rated as “yes” or “no”. The frequency of subjective side effects, such as nausea/vomiting, was also investigated. Cecal intubation rate and time were also investigated.

### 2.4. Statistical Analysis

The number of study subjects was calculated at a significance level of 0.025 (one-sided test), a power of 80%, a success rate of 85% (for both groups), a non-inferiority margin of 15%, and sample ratio of 1:1. Assuming a dropout rate of 3%, the number of patients required for the present study was 184.

Categorical variables are described as a number with a percentage, and continuous variables are expressed as a mean ± standard deviation (SD). An analysis of variance, Student’s *t*-test, and a chi-squared test were performed to examine differences between the groups. All reported P values were two-tailed, and differences with *p* ≤ 0.05 were considered to be statistically significant. Statistical analyses were performed using spreadsheet software (Excel 2010, Microsoft Corporation, Redmond, WA, USA) and SPSS version 24.0 (IBM Corp., Armonk, NY, USA) for Windows (Microsoft Corporation).

## 3. Results

### 3.1. Patient Characteristics

**Figure 1:**
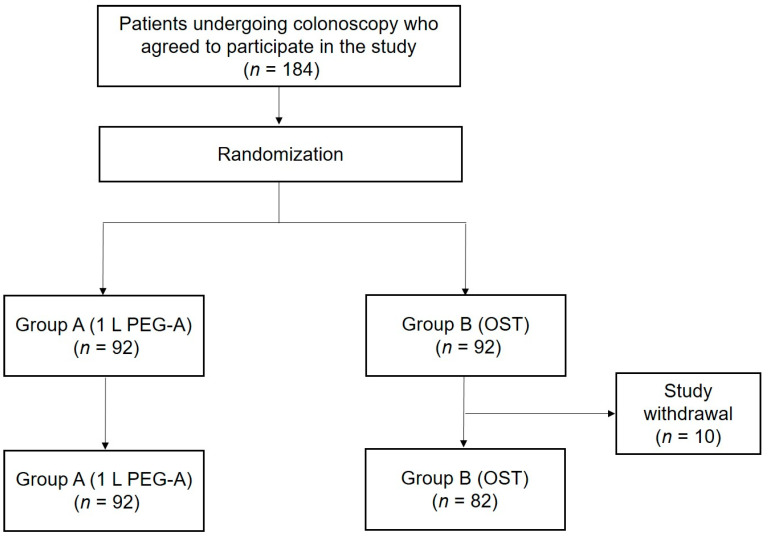
Flow diagram illustrating patient enrollment.

Of the 184 subjects initially enrolled, 10 from group B withdrew from the study. Included in the final analysis were 92 and 82 patients in groups A and B, respectively. The patients who withdrew from the study were those who postponed colonoscopy due to personal reasons or decided to undergo the procedure at another hospital. (Figure 1). The mean age of groups A and B was 59.9 and 57.2 years, respectively (*p* = 0.114). There were no statistical differences in all other baseline characteristics, including sex distribution, body mass index, and indications for colonoscopy, between the groups (Table 1).

### 3.2. Bowel Preparation Efficacy

**Figure 2:**
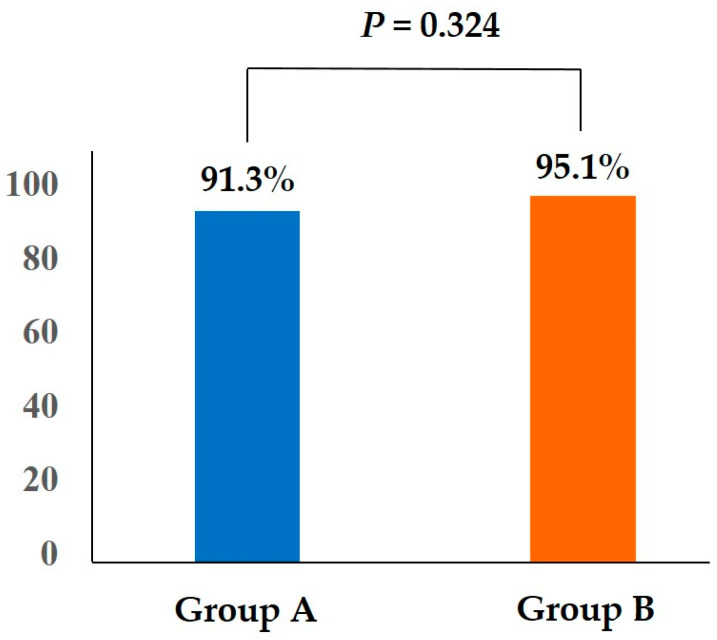
Bowel preparation success in both groups.

The total BBPS score was 6.5 ± 1.4 in group A and 7.1 ± 1.5 in group B (*p* = 0.011). There were no statistical differences in the proportion of adequate bowel preparations (BBPS score ≥2) in the right colon (93.5% vs. 95.1%; *p* = 0.642), the transverse, and the left colons (94.6% vs. 98.8% [*p* = 0.130]; 96.7% vs. 97.6% [*p* = 0.746]) or in the proportion of the total BBPS scores ≥6 (92.4% vs. 97.6%; *p* = 0.126) between groups A and B, respectively (Table 2). The proportion of bowel preparation success in group A was not inferior to that in group B (91.3% vs. 95.1%, *p* = 0.324, one-sided 97.5% lower confidence limit–5.4%) (Figure 2).

There were no patients in either group who failed cecal intubation. Cecal intubation time did not exhibit a significant difference between groups A and B (403.4 ± 270.0 s vs. 423.8 ± 302.0 s; *p* = 0.641).

### 3.3. Patient Tolerability and Safety

All patients in both groups completed taking the full dose of the designated preparation agents at least 2 h before colonoscopy. The mean overall satisfaction with the preparation was higher among those in group B vs. group A (8.2 ± 1.7 vs. 6.8 ± 2.0, respectively; *p* < 0.001). The proportion of participants willing to use the same preparation agent at next colonoscopy was also higher in group B vs. group A (73/82 [89.0%] vs. 65/92 [70.7%], respectively; *p* = 0.003).

The rate of overall adverse events was not different between groups A and B (27/92 [29.3%] vs. 21/82 [25.6%], respectively; *p* = 0.583). However, the frequency of abdominal distension was lower in group A vs. group B (3/92 [3.3%] vs. 9/82 [11.0%]; *p* = 0.045). The frequency of all other subjective discomforts, including nausea and vomiting, was not different between the two groups (Table 3). There were no serious complications in either group, and all patients who complained of adverse events improved without specific treatment.

### 3.4. Subgroup Analysis According to Patient Age

For the analysis according to a patient age of ≥65, there were 31 patients in group A, whereas there were 22 patients in group B. In the subgroup of patients ≥ 65 years of age, the proportion of successful bowel preparations (87.1% vs. 90.9%; *p* = 0.665), overall satisfaction with the preparation agent (7.4 ± 1.9 vs. 7.9 ± 1.9; *p* = 0.340), proportion of participants willing to use the same preparation agent at their next colonoscopy (22/31 [71.0%] vs. 19/22 [86.4%]; *p* = 0.194), and adverse events were not different between groups A and B, respectively (Table 4).

For the analysis according to a patient age of <65, there were 61 patients in group A, whereas there were 60 patients in group B. In the subgroup analysis of patients <65 years of age, there was no difference in the proportion of successful bowel preparations between groups A and B (93.4% vs. 96.7%; *p* = 0.417). Overall satisfaction was greater in group B vs. group A (6.5 ± 2.1 vs. 8.3 ± 1.6; *p* < 0.001). The proportion of participants willing to use the same preparation agent at their next colonoscopy was also greater in group B (43/61 [70.5%] vs. 54/60 [90.0%]; *p* = 0.007). Most adverse events occurred similarly in both groups, except for a slightly higher incidence of bowel distension in group B (Table 4).

## 4. Discussion

In this prospective, randomized, controlled trial, 1 L PEG-A and OSTs demonstrated similarly high efficacy in bowel preparation cleanliness, assessed according to the BBPS. OSTs demonstrated better tolerability, including superior overall satisfaction and higher willingness to repeat the same preparation at the next colonoscopy. Better tolerability to OSTs was evident in younger patients (i.e., <65 years of age), whereas the elderly (≥65 years of age) exhibited no difference in tolerability between 1 L PEG-A and OSTs. The safety profile was essentially equal between the two agents, except for more frequent abdominal distension in the OST group.

Inadequate bowel preparation can increase the risk for missed polyps, followed by interval cancer. Complete and safe endoscopic resection of colorectal polyps is also difficult if bowel preparation is poor, which may also hinder effective prevention of colorectal cancer. Therefore, the efficacy of bowel preparation agents is of utmost importance. Previous studies have reported a non-inferior preparation quality and adenoma detection rate with the 1 L PEG-A solution compared with the 2 L PEG-A solution and magnesium citrate plus sodium picosulfate [8,9]. The OST formulation has also demonstrated better bowel preparation efficacy than 2 L PEG-A [15]. In our study, although the total BBPS score was slightly better in the OST group, both the 1 L PEG-A and the OSTs, two relatively new bowel cleansing agents, demonstrated similar bowel preparation success rates of 91.3% and 95.1%, respectively, which meets the goal of bowel preparation adequacy (≥90%) for high-quality colonoscopy, as defined by the American Gastroenterological Association (AGA) [17]. This finding suggests that both the 1 L PEG-A and the OSTs can be used as reliable options for successful bowel preparation for colonoscopy. Successful cecal intubation may also reflect the adequate efficacy of bowel preparation agents because sufficient cleanliness is an important prerequisite for successful cecal intubation. The AGA suggests a cecal intubation rate goal of ≥90% for high-quality colonoscopy [17]. In our study, cecal intubation was successful in all patients within a reasonable average time of approximately 7 min in both groups, which, again, supports the excellent efficacy of both 1 L PEG-A and OSTs.

Tolerability was slightly better for OSTs compared with 1 L PEG-A, which was demonstrated by better overall satisfaction with the preparation and higher willingness to repeat the same preparation at the next colonoscopy in the OST group. The current 1 L PEG-A formulation is considered to have superior tolerability compared with conventional large-volume agents because of the low volume and improved taste and odor. Nevertheless, OSTs may be preferred because the odorless tablet formulation can be swallowed with water, preventing any manifestations of unpleasant taste and odor.

Safety was similarly acceptable in both the 1 L PEG-A and the OSTs, which was reflected by a similar rate of adverse events between the two groups and no need for specific treatment for any adverse events. The only adverse event with a difference was the more frequent development of abdominal distension in group B (i.e., OSTs). A previous study also reported a greater number of patients complaining of abdominal pain and distension in the OST group compared with the 2 L PEG group, although the difference was statistically insignificant [18]. The reason for more frequent abdominal distension with OST use remains unclear. However, we found some patients who complained of prolonged time to first stool passage after taking OSTs. They experienced abdominal distension before the first defecation. Unfortunately, we did not investigate the time between the administration of bowel preparation agents and the first bowel movement and its association with abdominal distension in detail; therefore, this should be analyzed in future studies. If the association among the time until the first defecation, abdominal distension, and OST is confirmed, further trials may be warranted to investigate the possibility of prokinetics as an adjunct to decrease the risk for abdominal distension caused by OST.

Older age has been reported to be a risk factor for poor bowel preparation [19]. In addition, adverse events, such as dehydration and electrolyte imbalance, may be more severe in those with advanced age. Therefore, bowel preparations for elderly patients should be more efficacious and safer. In our subgroup analysis, bowel preparation success rates for the 1 L PEG-A and OST groups were >85%, and the total BBPS score ≥6 was >90% in both groups, demonstrating high efficacy in older patients (i.e., ≥65 years). In addition, tolerability and safety parameters were numerically similar to those of younger patients. Interestingly, unlike younger patients (<65 years), who exhibited better tolerability to OSTs, older patients (≥65 years) exhibited no difference in overall satisfaction between 1 L PEG-A and OSTs and reported the same willingness to use the same agents at the next colonoscopy. The OST preparation used in this study measured 17.2 × 8.2 × 6.4 mm in size. The elderly may find it difficult to swallow many large tablets, unlike younger patients, which may be a reason for the absence of preference for OSTs. Because both agents demonstrated efficacy, tolerability, and safety and no specific preference was evident in older patients, we suggest that either the 1 L PEG-A or the OSTs can be offered as an effective option for bowel preparation for colonoscopy in the elderly patient population.

Our study had several limitations, the first of which was its single tertiary center design, which may limit the generalizability of the results; as such, additional validation is necessary in multicenter studies. Second, safety data were acquired only through questionnaires. Laboratory investigations should be performed for more objective monitoring of safety, especially in the elderly group. Third, although the sample size was derived from statistical calculations, the relatively small number of patients enrolled in each group is a limitation of this study. In particular, the small number of older patients may lower the confidence of findings of the analyses. Future studies should enroll a larger number of patients. Specifically, studies involving a greater number of older patients are needed to reliably confirm the adequacy of 1 L PEG-A vs. OSTs in the elderly. Finally, although no differences were observed between the two groups regarding chronic constipation, history of abdominal surgery, and use of antidepressants, we could not determine whether there was a difference between the groups in the frequency of inappropriate bowel preparation, another risk factor for poor bowel preparation, during the previous colonoscopy.

In conclusion, both the 1 L PEG-A and the OSTs were effective, tolerable, and safe for bowel preparation. The OST formulation was found to be slightly better tolerated, whereas the 1 L PEG-A solution resulted in a lower frequency of abdominal distension. Nevertheless, both agents were effective and acceptable in older patients.

## Figures and Tables

**Table 1:** Baseline characteristics of patients.

Characteristics	Group A (n = 92)	Group B (n = 82)	*p* Value
Age (years), mean ± SD	59.9 ± 10.6	57.2 ± 11.6	0.114
Sex (%)			0.410
Male	54 (58.7%)	43 (52.4%)	
Female	38 (41.3%)	39 (47.6%)	
Height (cm), mean ± SD	164.4 ± 7.8	164.9 ± 8.2	0.685
Weight (kg), mean ± SD	67.6 ± 11.4	66.5 ± 12.8	0.578
BMI (kg/m^2^), mean ± SD	24.9 ± 3.1	24.3 ± 3.3	0.221
Indication of colonoscopy			0.322
Screening/surveillance	84 (91.3%)	71 (86.6%)	
Diagnostic	8 (8.7%)	11 (13.4%)	
Chronic constipation (%)	10 (10.9%)	8 (9.8%)	0.995
History of abdominal surgery (%)	19 (20.7%)	19 (23.2%)	0.768
Use of narcotics/antidepressants (%)	3 (3.3%)	2 (2.4%)	0.508

BMI, body mass index; SD, standard deviation.

**Table 2:** Comparison of bowel preparation efficacy.

	Group A (n = 92)	Group B (n = 82)	*p* Value
Bowel preparation success	84 (91.3%)	78 (95.1%)	0.324
BBPS score ≥ 2 in the right colon, *n* (%)	86 (93.5%)	78 (95.1%)	0.642
BBPS score ≥ 2 in the transverse colon, *n* (%)	87 (94.6%)	81 (98.8%)	0.130
BBPS score ≥ 2 in the left colon, *n* (%)	89 (96.7%)	80 (97.6%)	0.746
BBPS score ≥ 6 in total, *n* (%)	85 (92.4%)	80 (97.6%)	0.126

BBPS, Boston Bowel Preparation Scale.

**Table 3:** Patient satisfaction, acceptability, and safety.

	Group A (n = 92)	Group B (n = 82)	*p* Value
Overall satisfaction with the preparation (0–10), mean ± SD	6.8 ± 2.0	8.2 ± 1.7	<0.001
Willingness to repeat the same preparation at next colonoscopy, n (%)	65 (70.7%)	73 (89.0%)	0.003
Adverse events, *n* (%)	27 (29.3%)	21 (25.6%)	0.583
Abdominal distension, *n* (%)	3 (3.3%)	9 (11.0%)	0.045
Abdominal pain, *n* (%)	1 (1.1%)	2 (2.4%)	0.506
Nausea, *n* (%)	23 (25.0%)	14 (17.1%)	0.204
Vomiting, *n* (%)	8 (8.7%)	4 (4.9%)	0.324
Chilling sensation, *n* (%)	1 (1.1%)	2 (2.4%)	0.506
Dizziness, *n* (%)	3 (3.3%)	6 (7.3%)	0.320
Headache, *n* (%)	1 (1.1%)	1 (1.2%)	0.935
Hoarseness, *n* (%)	1 (1.1%)	0 (0.0%)	0.347

**Table 4:** Comparison of bowel preparation efficacy, tolerability and safety between 1 L PEG-A and OSTs in patients <65 years and ≥65 years.

	Patients <65 Years			Patients ≥65 Years		
	Group A (n = 61)	Group B (n = 60)	*p* Value	Group A (n = 31)	Group B (n = 22)	*p* Value
Bowel preparation success	57 (93.4%)	58(96.7%)	0.417	27 (87.1%)	20 (90.9%)	0.665
BBPS score ≥ 2 in the right colon, *n* (%)	58 (95.1%)	58 (96.7%)	0.664	28 (90.3%)	20 (90.9%)	0.944
BBPS score ≥ 2 in the transverse colon, *n* (%)	57 (93.4%)	59 (98.3%)	0.179	30 (96.8%)	22 (100.0%)	0.325
BBPS score ≥ 2 in the left colon, *n* (%)	59 (96.7%)	59 (98.3%)	0.571	30 (96.8%)	21 (95.5%)	0.814
BBPS score ≥ 6 in total, *n* (%)	57 (93.4%)	59 (98.3%)	0.179	28 (90.3%)	21 (95.5%)	0.470
Overall satisfaction with the preparation (0–10), mean ± SD	6.5 ± 2.1	8.3 ± 1.6	<0.001	7.4 ± 1.9	7.9 ± 1.9	0.340
Willingness to repeat the same preparation at next colonoscopy, n (%)	43 (70.5%)	54 (90.0%)	0.007	22 (71.0%)	19 (86.4%)	0.194
Adverse events, *n* (%)	18 (29.5%)	16 (26.7%)	0.731	9 (29.0%)	5 (22.7%)	0.612
Abdominal distension, *n* (%)	1 (1.6%)	6 (10.0%)	0.049	2 (6.5%)	3 (13.6%)	0.146
Abdominal pain, *n* (%)	0 (0.0%)	1 (1.7%)	0.315	1 (3.2%)	1 (4.5%)	0.814
Nausea, *n* (%)	16 (26.2%)	11 (18.3%)	0.301	7 (22.6%)	3 (13.6%)	0.407
Vomiting, *n* (%)	7 (11.5%)	2 (3.3%)	0.089	1 (3.2%)	2 (9.1%)	0.412
Chilling sensation, *n* (%)	1 (1.6%)	2 (3.3%)	0.554	0 (0.0%)	0 (0.0%)	-
Dizziness, *n* (%)	3 (4.9%)	5 (8.3%)	0.455	0 (0.0%)	1 (4.5%)	0.239
Headache, *n* (%)	58 (95.1%)	58 (96.7%)	0.664	0 (0.0%)	0 (0.0%)	-
Hoarseness, *n* (%)	1 (1.6%)	1 (1.7%)	0.991	1 (3.2%)	0 (0.0%)	0.325

## Data Availability

Data are contained within the article.
